# Maternal use of antibiotics and cancer incidence risk in offspring: A population‐based cohort study in Manitoba, Canada

**DOI:** 10.1002/cam4.2412

**Published:** 2019-07-16

**Authors:** Xibiao Ye, Barret A. Monchka, Christiaan H. Righolt, Salaheddin M. Mahmud

**Affiliations:** ^1^ Vaccine and Drug Evaluation Centre University of Manitoba Winnipeg Manitoba Canada; ^2^ School of Health Information Science University of Victoria British Columbia Canada; ^3^ College of Pharmacy University of Manitoba Winnipeg Manitoba Canada; ^4^ George & Fay Yee Centre for Healthcare Innovation University of Manitoba Winnipeg Manitoba Canada

**Keywords:** antibiotics, childhood cancer, Prenatal exposure, trimester

## Abstract

Several epidemiological studies have found an association between maternal antibiotics use during pregnancy and increased risk of certain cancer types, although conclusions differ between studies. We examined this association in a cohort study including 262 116 mother‐child pairs of Manitoba births between 1996 and 2013. Maternal antibiotics use during prepregnancy (6 months prior to pregnancy) and pregnancy periods was assessed. Children's cancer incidence was tracked up to the end of the follow‐up period (December 2015). We calculated incidence rate and used Cox regression to estimate adjusted hazard ratios (HRs). Antibiotics use during pregnancy was not associated with overall cancer (HR = 1.1, 95% confidence interval 0.9‐1.4), leukemias (1.3, 0.9‐1.8), or acute lymphocytic leukemia (1.1, 0.7‐1.6). The association between antibiotics use and overall cancer risk differed by trimester: 1.5 (1.1‐1.9) in the first, 0.8 (0.6‐1.0) in the second, and 1.1 (0.8‐1.5) in the third trimester. Further research is necessary to confirm the association between first‐trimester exposure and cancer risk after a better controlling of confounding factors.

## 1 INTRODUCTION

Prescription drug use during pregnancy is common and increasing. Around 60% of pregnant women in British Columbia of Canada received at least one prescription in 2002 (a median of two prescriptions); this proportion increased to 66% by 2011 (the median number of prescriptions rose to 3).^1^ A quarter of pregnant women received prescriptions across all trimesters.^1^ Similar increasing trends were reported in other countries.^2^, ^3^ Antibiotics are the most commonly used medications by pregnant women,^4^ but pregnant women and children are often excluded from clinical trials due to safety concerns. Many drugs and/or their metabolites can cross the placenta barrier, causing in utero exposures.^5^ Postmarketing surveillance typically focuses on pregnancy outcomes (eg, fetal loss, preterm birth) and birth defects and rarely on the long‐term effects (eg, cancer and chronic diseases) in offspring.

Several studies indicated that use of antibiotics and other anti‐infectives increased the risk of some cancers in offspring. This association was reported for acute lymphocytic leukemia (ALL), acute myeloid leukemia (AML), Burkitt lymphoma, noncentral nervous system tumors, and rhabdomyosarcoma,^6^, ^7^ although other studies did not report these associations.^8^ A recent study found that the lower risk of AML among children was associated with their mothers' antibiotics use during pregnancy.^9^ The number of epidemiologic studies of this relation is limited and the findings have been inconsistent. Most studies relied on patient's self‐reported information on drug use, which is prone to recall bias and misclassification.^10^ Fewer studies have examined the effects of exposure timing on cancer risk in offspring. We linked administrative databases with a cancer registry to study the association between maternal use of antibiotics and cancer risk in offspring.

## 2 METHODS

### 2.1 Data sources

Manitoba Health (MH) is the publicly funded health insurance agency providing comprehensive health insurance, including coverage for hospital and outpatient physician services, to the province's 1.3 million residents. Coverage is universal with no eligibility distinction based on age or income, and participation rates are very high (>99%).^11^ Insured services include hospital, physician, and preventive services including vaccinations. MH maintains several centralized, administrative electronic databases that are linkable using a unique personal health identification number (PHIN). The completeness and accuracy of MH administrative databases are well established.^12^, ^13^ These databases have been used extensively in studies of disease surveillance and postmarketing evaluation of various vaccines and drugs.^14^, ^15^, ^16^

The MH Population Registry tracks addresses and dates of birth, death, and insurance coverage for all insured persons. The Hospital Discharge Abstract Database recorded virtually all services provided since 1971 by hospitals in the province, using the International Classification of Diseases, Tenth Revision, Canadian Edition (ICD‐10‐CA) since 2004, including admissions and day surgeries. The Medical Services Database, also in operation since 1971, collects similar information, based on physician fee‐for‐service or shadow billing, on services provided by physicians in offices, hospitals, and outpatient departments across the province. The Drug Program Information Network (DPIN) captures data from pharmacy claims since 1995 for formulary drugs dispensed to all Manitobans even those without prescription drug coverage. CancerCare Manitoba maintains one of the oldest population‐based cancer registries in the world (MCR, in operation since 1956). Reporting of cancer cases is mandated by provincial regulations and required for payments of physicians’ service claims. The MCR is regularly audited by the North American Association of Central Cancer Registries and the quality of cancer registration has been consistently very high.^13^ Most cases are pathologically confirmed (94% for cases registered between 2003 and 2007) and less than 2% of registrations originate from death certificates.^13^ The Families First Screen (formerly the Baby First Screen), in existence since 2002, includes measures of substance uses, mental health, pregnancy complications, and financial difficulties for mothers of newborns in Manitoba evaluated during postpartum visits by public health nurses.

### 2.2 Study design

We conducted a retrospective cohort study and included all children who (a) were born in Manitoba between 1 January 1996 and 31 December 2013, and (b) had MH coverage at birth. Children with diagnosed chromosomal abnormalities (defined as one hospitalization or two physician visits with ICD‐10‐CA code Q90‐Q99 or ICD‐9‐CM code 758) were excluded.

The exposure was maternal antibiotics use (Anatomical Therapeutic Chemical code J01) as determined from the DPIN. We measured maternal drug use during pregnancy (prenatal exposure) as well as during the 6‐month period before pregnancy (prepregnancy exposure). Prenatal use was further classified by trimester (gestational day 1‐90, 91‐195, and 196 to birth). The first day of the woman's last menstrual period was determined as the first day of gestation.

Follow‐up started at birth and ended at the earliest of the date of diagnosis of a first primary cancer (excluding nonmelanoma skin cancer), loss of MH coverage for any reason (including migration and death), or 31 December 2015 (the end of the follow‐up period). We identified all cancer diagnoses, classified using the International Classification of Childhood Cancer, 3rd Edition (ICCC‐3), by linkage with the MCR.^17^

### 2.3 Statistical analysis

We calculated the crude cancer incidence rates (per 100 000 person‐years) and 95% confidence intervals (95% CIs). We compared the incidence rates for those exposed and unexposed using crude incidence rate ratios (IRRs). We used proportional hazards model (Cox regression) to estimate adjusted hazard ratios (HRs) and 95% CIs separately for prenatal and prepregnancy exposure. Based on clinical significance or a change in estimates over 5% after adjustment, we adjusted all models for maternal age, neighborhood income quintile, and prenatal substance use (tobacco, alcohol, or illicit drug use as recorded in the Families First Screen). We verified that none of these variables was an effect modifier. To estimate the effect of timing of prenatal use, we repeated the analysis separately for each trimester while adjusting for use in other trimesters. We repeated all analyses for the most frequently diagnosed cancers: all leukemias and ALL (we could not do the same for other cancer types due to the small number of cases). Studies indicate that prenatal exposure might be associated with a peak in cancer risk at early ages (<5 years) but not later.^18^, ^19^ We analyzed cancers diagnosed within age of 5 years and within age of 19 years separately.

## 3 RESULTS

The full birth cohort comprised 262 972 children, 856 of them were excluded because they were born with chromosomal abnormalities. Of the remaining 262 116 children, 38% had a history of maternal antibiotics use (Table 1). Exposed and unexposed cohorts had similar distributions of gender, size‐for‐gestational‐age, and number of children in household. Exposed children were more likely to live in lower income communities, be born to younger mothers, and be born to mothers with reported substance use.

**Table 1:** Number (%) of mothers using antibiotics during pregnancy according to certain socioeconomical and clinical characteristics

Characteristic	Maternal antibiotics use during pregnancy	
	Yes (N = 98 997)	No (N = 163 119)
Child gender		
Male	50 918 (51.4%)	83 592 (51.2%)
Female	48 079 (48.6%)	79 527 (48.8%)
Place of residence		
Urban	51 732 (52.3%)	91 652 (56.2%)
Rural	45 321 (45.8%)	67 893 (41.6%)
Unknown	1944 (2.0%)	3574 (2.2%)
Income quintile		
Q1 (lowest)	30 398 (30.7%)	38 450 (23.6%)
Q2	20 450 (20.7%)	34 488 (21.1%)
Q3	17 662 (17.8%)	29 781 (18.3%)
Q4	15 621 (15.8%)	29 888 (18.3%)
Q5 (highest)	12 922 (13.1%)	26 938 (16.5%)
Unknown	1944 (2.0%)	3574 (2.2%)
Size for gestational age		
Small for gestational age	7432 (7.5%)	12 685 (7.8%)
Appropriate for gestational age	76 572 (77.3%)	128 182 (78.6%)
Large for gestational age	14 949 (15.1%)	22 146 (13.6%)
Unknown	44 (< 0.1%)	106 (0.1%)
Maternal age (years)		
<20	11 294 (11.4%)	12 541 (7.7%)
20‐24	24 882 (25.1%)	32 265 (19.8%)
25‐29	28 967 (29.3%)	50 027 (30.7%)
30‐34	22 806 (23.0%)	45 634 (28.0%)
35+	11 048 (11.2%)	22 652 (13.9%)
Number of children in household		
1	43 766 (44.2%)	76 734 (47.0%)
2	29 999 (30.3%)	49 690 (30.5%)
3	14 457 (14.6%)	21 302 (13.1%)
4+	10 775 (10.9%)	15 393 (9.4%)
Mother had cancer	4359 (4.4%)	5943 (3.6%)
Mother's substance use^a^		
Yes	17 867 (18.0%)	19 440 (11.9%)
No	36 659 (37.0%)	72 791 (44.6%)
Unknown	44 471 (44.9%)	70 888 (43.5%)

a Any of tobacco, alcohol, or illicit drugs

During the follow‐up, 361 children (0.1%) were diagnosed with cancer (Table 2). The incidence rate of overall cancer was 14 per 100 000 person‐years (95% CI 12‐17) for children under 20 years exposed to antibiotics during pregnancy and 13 (11‐15) for those who were not, resulting in a crude IRR of 1.1 (0.9‐1.4). The IRR was 1.3 (0.9‐1.8) for leukemia and 1.1 (0.7‐1.6) for ALL during the full follow‐up (Table 2). For all cancers as well as leukemias and ALL, the IRR was higher for use in the first trimester, 1.4 (1.0‐1.8) compared to 0.8 (0.6‐1.1) and 1.1 (0.8‐1.4) for the second and third trimester, respectively. The IRRs were generally similar when limiting the follow‐up to 5 years (Supplementary table 1).

**Table 2:** Crude incidence rates (per 100 000 person‐years) and incidence rate ratios (95% confidence interval) of the association between maternal antibiotics use and childhood cancer

Time of exposure	Exposed	Overall			Leukemias, myeloproliferative diseases, and myelodysplastic diseases			ALL		
		Cases	Incidence rate	Incidence rate ratio	Cases	Incidence rate	Incidence rate ratio	Cases	Incidence rate	Incidence rate ratio
Prepregnancy	Yes	88	12 (10‐15)	0.8 (0.7‐1.1)	42	6 (4‐8)	1.1 (0.8‐1.6)	34	5 (3‐6)	1.1 (0.7‐1.7)
	No	273	14 (12‐16)		99	5 (4‐6)		80	4 (3‐5)	
Anytime during pregnancy	Yes	145	14 (12‐17)	1.1 (0.9‐1.4)	62	6 (5‐8)	1.3 (0.9‐1.8)	46	5 (3‐6)	1.1 (0.7‐1.6)
	No	216	13 (11‐15)		79	5 (4‐6)		68	4 (3‐5)	
First trimester	Yes	76	17 (14‐22)	1.4 (1.0‐1.8)	30	7 (5‐10)	1.4 (0.9‐2.1)	23	5 (3‐8)	1.3 (0.8‐2.1)
	No	285	13 (11‐14)		111	5 (4‐6)		91	4 (3‐5)	
Second trimester	Yes	60	12 (9‐15)	0.8 (0.6‐1.1)	32	6 (4‐9)	1.2 (0.8‐1.9)	22	4 (3‐7)	1.0 (0.6‐1.6)
	No	301	14 (12‐16)		109	5 (4‐6)		92	4 (3‐5)	
Third trimester	Yes	61	14 (11‐19)	1.1 (0.8‐1.4)	26	6 (4‐9)	1.2 (0.8‐1.9)	19	5 (3‐7)	1.1 (0.6‐1.8)
	No	300	13 (12‐15)		115	5 (4‐6)		95	4 (3‐5)	

Abbreviation: ALL, acute lymphocytic leukemia.

In multivariate analyses (Table 3), antibiotics use during pregnancy was not associated with the risk of overall cancer (HR = 1.1, 0.9‐1.4), leukemias (HR = 1.3, 0.9‐1.8), or ALL (HR = 1.1, 0.7‐1.6). First trimester exposure was associated with a higher risk of overall cancer (HR = 1.5, 1.1‐1.9) for children under 20 years and for children under 5 years (HR = 1.5, 1.0‐2.0). The HR was also elevated for leukemias and ALL. Antibiotics use during the second and third trimesters was not associated with cancer risk. Prepregnancy use was not associated with increased cancer risk (Table 3).

**Table 3:** Adjusted hazard ratios^a^ (95% confidence interval) of the association between maternal antibiotics use and childhood cancer

Time of exposure	Under 5 y			Full follow‐up		
	Any cancer	Leukemias, myeloproliferative diseases, and myelodysplastic diseases	ALL	Any cancer	Leukemias, myeloproliferative diseases, and myelodysplastic diseases	ALL
Prepregnancy	0.9 (0.6‐1.2)	1.0 (0.6‐1.5)	1.0 (0.6‐1.6)	0.9 (0.7‐1.1)	1.1 (0.8‐1.6)	1.1 (0.7‐1.7)
Anytime during pregnancy	1.0 (0.8‐1.3)	1.1 (0.7‐1.7)	1.0 (0.6‐1.6)	1.1 (0.9‐1.4)	1.3 (0.9‐1.8)	1.1 (0.7‐1.6)
First trimester^b^	1.5 (1.0‐2.0)	1.5 (0.9‐2.5)	1.5 (0.9‐2.6)	1.5 (1.1‐1.9)	1.3 (0.9‐2.0)	1.3 (0.8‐2.1)
Second trimester^b^	0.6 (0.4‐0.9)	0.9 (0.5‐1.5)	0.8 (0.4‐1.5)	0.8 (0.6‐1.0)	1.1 (0.7‐1.7)	0.9 (0.6‐1.5)
Third trimester^3^	1.1 (0.7‐1.6)	0.9 (0.5‐1.6)	0.8 (0.4‐1.6)	1.1 (0.8‐1.5)	1.1 (0.7‐1.7)	1.0 (0.6‐1.7)

Abbreviation: ALL, acute lymphocytic leukemia,

a Adjusted for prenatal substance use (any of tobacco, alcohol, or illicit drugs), maternal age, and income quintile.

b Also adjusted for exposure in other trimesters

## 4 DISCUSSION

We found that maternal antibiotics use during the first trimester was associated with a higher risk of overall cancer among children. However, prenatal antibiotics uses during other time windows (before pregnancy and during the second and third trimesters) were not associated with overall cancer risk among offspring. Antibiotics use was not associated with the risk of leukemias or ALL.

Previous studies reported mixed results. Maternal antibiotics use was either associated with an increased risk of childhood cancers, in particular leukemia, or no association was found. Maternal antibiotics use ascertained by maternal interviews has been associated with an increased risk of all childhood cancer in Quebec (age under 10, odds ratio [OR] = 1.50 [1.02‐2.21])^18^ and Germany (age under 15, OR = 1.42 [1.08‐1.87]).^6^ A Danish‐Swedish cohort study found no association with exposure anytime during pregnancy (age under 15, HR = 1.08 [0.96‐1.18]), only during the first or second trimester, but a slightly increased risk with exposure only in the third trimester (HR = 1.30 [1.06‐1.58]).^9^ Leukemia risk was not associated with maternal use of antibiotics in a Scottish case‐control study based on interviews,^20^ a Danish case‐control study using administrative data,^8^ or a Danish‐Swedish cohort study.^9^ Infant acute leukemias (ALL and AML combined) were not associated with maternal amoxicillin or ciprofloxacin use in Brazil.^21^ An increased ALL risk has been associated with maternal antibiotics use in some of the aforementioned studies,^9^, ^18^, ^20^ although other studies reported no association.^6^, ^7^, ^8^

Some of the previous studies were limited due to (structured) interviews. Differential misclassification of the exposure (self‐reported antibiotics use might be subject to a recall bias away from the null^10^) in those studies could have biased the association. Most other studies employed a case‐control design, whereas our study and Momen et al^9^ used a cohort design. Our results for the prepregnancy and pregnancy periods are in line with Momen et al They limited their analysis to exposure in specific trimesters, whereas we adjusted use in each trimester for use in the other trimesters.

A major strength of this study is the availability of high‐quality and population‐based health administrative databases in Manitoba. The completeness and accuracy of the MCR and MH databases are well established.^12^, ^13^ Misclassification of childhood cancer is rare; it is typically a severe, symptomatic disease and provincially mandated to be reported to the MCR. Detection bias cannot be ruled out as children exposed to antibiotics prenatally are more likely to seek heath care due to increased risk of diseases such as asthma.^22^ Loss to follow‐up likely caused bias but the magnitude might be small as the follow‐up rate was high and there is no solid evidence on the correlation between maternal antibiotics use and the reasons for loss to follow‐up. Misclassification of maternal antibiotics use is also rare as DPIN is a central database that records pharmacy dispensations (the typical source of prescription antibiotics) to all Manitobans. We lacked information on hospital‐dispensed antibiotics, which might have resulted in misclassification of exposure. Another possible source of exposure misclassification is compliance (ie, patients might have not used prescribed antibiotics). Other potential confounders such as occupational and environmental exposures and tobacco use during pregnancy have not been adjusted for.

It is biologically implausible that all antibiotics cause all types of cancers. In a Danish‐Swedish study, increased cancer risk among children under 5 years was associated with the use of specific antibiotics such as ciprofloxacin, pivampicillin, and phenoxymethylpenicillin.^9^ The study found an association between prenatal antibiotics use and risk of leukemias, but not other cancer types.^9^ Momen et al reported an inverse association between prenatal antibiotics exposure and a lower risk of AML.^9^ It is unclear where this could be explained by the hygiene hypothesis which primarily focuses on infection during the first year of life.^23^ Analysis by subtype would have provided a clearer picture and could have provided more evidence for specific follow‐up studies, but we were underpowered to limit our analysis to specific antibiotics or cancer subtypes other than leukemia and ALL.

In conclusion, the observed association in the first trimester may be due to uncontrolled confounding. The role of chance or spurious finding cannot be ruled out. More research is warranted to assess the effect of timing of antibiotics use during pregnancy on the risk of childhood cancer.

## CONFLICT OF INTEREST

SMM has received unrestricted research grants from GlaxoSmithKline, Merck, Sanofi Pasteur, Pfizer, and Roche‐Assurex for unrelated studies. None of the other authors has any conflicts of interest that could affect the design or analysis of this project.

## AUTHORS’ CONTRIBUTION

XY and SMM designed and supervised the study, BAM and CHR analyzed the data, XY and CHR wrote the manuscript. All authors contributed to interpretation of the results, critically revised the manuscript and approved the final draft for submission.

## DATA SHARING

Data used in this article were derived from administrative health and social data as a secondary use. The data were provided under specific data sharing agreements only for approved use at MCHP. The original source data are not owned by the researchers or Manitoba Centre for Health Policy (MCHP) and as such cannot be provided to a public repository. The original data source and approval for use have been noted in the acknowledgments of the article. Where necessary, source data specific to this article or project may be reviewed at MCHP with the consent of the original data providers, along with the required privacy and ethical review bodies.

## Data Availability

The data was provided under specific data sharing agreements only for approved use at MCHP. The original source data is not owned by the researchers or Manitoba Centre for Health Policy (MCHP) and as such cannot be provided to a public repository. The original data source and approval for use has been noted in the acknowledgments of the article. Where necessary, source data specific to this article or project may be reviewed at MCHP with the consent of the original data providers, along with the required privacy and ethical review bodies.
