# Supplementary material for: Maternal use of antibiotics and cancer incidence risk in offspring: A population‐based cohort study in Manitoba, Canada
Source: Cancer Med. 2019 Jul 16;8(11):5367–72. doi: 10.1002/cam4.2412 (PMC6718549; doi:10.1002/cam4.2412)
Supplement: Supplementary file 1 [file CAM4-8-5367-s001.docx]

**Maternal Use of Antibiotics and Cancer Incidence Risk in Offspring: A Population-based Cohort Study in Manitoba, Canada**

Xibiao Ye, Barret A. Monchka, Christiaan H. Righolt, Salaheddin M. Mahmud

Supplementary table 1: Crude incidence rates (per 100,000 person-years) and incidence rate ratios (95% confidence interval) of the association between maternal antibiotic use and childhood cancer before 5 years of age

| **Time of exposure** | **Exposed** | **Overall** | | | **Leukemias, myeloproliferative diseases, and myelodysplastic diseases** | | | **ALL^1^** | | |
| --- | --- | --- | --- | --- | --- | --- | --- | --- | --- | --- |
|  |  | **Cases** | **Incidence rate** | **Incidence rate ratio** | **Cases** | **Incidence rate** | **Incidence rate ratio** | **Cases** | **Incidence rate** | **Incidence rate ratio** |
| Pre-pregnancy | Yes | 55 | 17 (13-22) | 0.9 (0.6-1.2) | 25 | 8 (5-11) | 1.0 (0.6-1.6) | 21 | 6 (4-10) | 1.0 (0.6-1.6) |
|  | No | 166 | 19 (17-22) |  | 67 | 8 (6-10) |  | 57 | 7 (5-9) |  |
| Anytime during pregnancy | Yes | 83 | 18 (15-23) | 1.0 (0.7-1.3) | 38 | 8 (6-12) | 1.2 (0.7-1.8) | 31 | 7 (5-10) | 1.1 (0.7-1.7) |
|  | No | 138 | 19 (16-22) |  | 54 | 7 (6-10) |  | 47 | 6 (5-8) |  |
| 1st trimester | Yes | 46 | 23 (17-31) | 1.3 (0.9-1.8) | 21 | 11 (7-16) | 1.5 (0.9-2.5) | 17 | 9 (5-14) | 1.4 (0.8-2.4) |
|  | No | 175 | 18 (15-20) |  | 71 | 7 (6-9) |  | 61 | 6 (5-8) |  |
| 2nd trimester | Yes | 32 | 14 (10-20) | 0.7 (0.5-1.0) | 18 | 8 (5-12) | 1.0 (0.6-1.7) | 14 | 6 (4-10) | 0.9 (0.5-1.7) |
|  | No | 189 | 20 (17-23) |  | 74 | 8 (6-10) |  | 64 | 7 (5-8) |  |
| 3rd trimester | Yes | 35 | 19 (14-27) | 1.0 (0.7-1.5) | 14 | 8 (5-13) | 1.0 (0.5-1.8) | 11 | 6 (3-11) | 0.9 (0.4-1.7) |
|  | No | 186 | 18 (16-21) |  | 78 | 8 (6-10) |  | 67 | 7 (5-8) |  |
| *^1^ ALL = acute lymphocytic leukemia* | | | | | | | | | | |
